# Fever Nursing

**Published:** 1908-03-21

**Authors:** 


### Nursing Administration.

### FEVER NURSING.

### III.—THE NEW ASSOCIATION OF FEVER NURSES.

FEVER nursing is now a branch of the municipal service in which thousands of nurses are engaged, but hitherto these nurses have had no organisation of their own, and no means of safeguarding their common interests. Their work is becoming highly specialised, particularly on the preventive side, and they naturally regard their special training as an asset, one which would be greatly reduced in value if State Registration were adopted and fever training were not recognised in the system of registration. Hence this anxiety as to their position under State Registration which is the main motive force behind the organisation of fever nurses now taking shape. It is not that fever nurses are in general antagonistic to State Registration. Like some other trained nurses, they may be strongly in favour of it, owing to the impression that has been sedulously fostered for many years that registration will at once and for ever sweep away the competition of untrained women and all the difficulties under which nursing is at present carried on. We cannot, however, endorse this opinion, nor could we concur in any proposition to recognise the special training of fever nurses under any system of registration, in a separate section, unless this training was treated as an adjunct to three years' regular training in a general hospital.

In 1898, at the time when the Royal British Nurses' Association was practically reconstituted under a new set of by-laws, a vigorous attempt was made to secure recognition for fever nurses in a separate List of Nurses registered by that Association. Although that effort was unsuccessful, the training of fever nurses who have had three years' general training has been granted recognition under the new scheme of nursing diplomas initiated by the Royal British Nurses' Association. This is, so far as we are aware, the only instance in which the status of the fever nurse has been recognised in the setting of a general nursing curriculum.

The objects of the Fever Nurses' Association, as at present defined, are, in the first place, to standardise fever training, and this in respect both of the period of training and the quality of the instruction given. At present a matron who desires to obtain fever training for any of her nurses has no means, except private inquiry, of knowing in what hospitals really satisfactory training is provided. The Association will lay down certain conditions under which they will recognise fever hospitals as entitled to take probationers or give supplementary courses of training to nurses already trained in general hospitals. It may be said that the time is ripe for the development of this part of the scheme. Much difficulty is experienced in fever hospitals in obtaining the right kind of probationers—such as are capable of turning into satisfactory nurses—and in marking out defined conditions which shall entitle fever institutions offering adequate facilities to rank as training schools. The Association will put within the reach of all a powerful method of attracting earnest-minded women to this work. It will be a great gain also to have some pronouncement on the length of time which should constitute fever training. The hospital-trained nurse is too often contented to pass a mere three months in gaining fever "experience." Bearing in mind that for the first month she is almost as useless as a raw probationer, so different are the conditions under which she works, it is clear that three months is hardly sufficient to teach her the A B C of infectious nursing. It seems probable that the Fever Nurses' Association will set the standard at a year's training, at least, for those nurses trained in general hospitals who desire to gain a fever nurse's certificate, this year to be taken, however, if circumstances admit, as part of the whole three years' training. It is hoped to obtain some form of recognition, at present not fully defined, for the large class of fever nurses who have obtained fever training only. It is believed that a useful purpose might be served in organising this somewhat heterogeneous body of workers by selecting such as are skilled in their own kind of work, and placing their names on a separate register. They might at least be kept together to serve as a reserve force to draw upon in times of epidemic.

Among other aims of the new association, it is proposed to establish a supplementary examination and certificate for fever nurses, to appoint an educational committee with this object in view, to organise, if possible, some system of defence for nurse members in the exercise of their calling, and to use every means to awaken a spirit of reform in institutions now lagging so far behind in details of internal management as to constitute a distinct danger to persons seeking employment.

It seems tolerably certain that the new association will have the strength of unanimity in its policy, and under such conditions it will doubtless wield some power in bringing its policy to a practical issue. We commend it to the attention of medical officers and all superintendents of nursing in infectious institutions throughout the country. Too often it happens that movements in favour of the amelioration of the condition of workers fail to achieve their full aims on account of the preponderance of a waiting attitude among those chiefly concerned. This, we fear, is particularly the case among matrons and nurses in general. They wait to see what personal benefit they are likely to obtain themselves from co-operation, and forget that what benefits all, must in the nature of things benefit in the long run also the individual. A heavy responsibility rests on members of a profession when they omit to do their little part in furthering the aims of an organisation founded directly in their own interests.

